# Production of biologically active human interleukin-10 by *Bifidobacterium bifidum* BGN4

**DOI:** 10.1186/s12934-020-01505-y

**Published:** 2021-01-19

**Authors:** Nayoun Hong, Seockmo Ku, Kyungjin Yuk, Tony V. Johnston, Geun Eog Ji, Myeong Soo Park

**Affiliations:** 1 Department of Food and Nutrition, Research Institute of Ecology, SeoulNationalUniversity, Seoul, 08826 Korea; 2 Fermentation Science Program, School of Agriculture, College of Basic and Applied Sciences, Middle Tennessee State University, Murfreesboro, TN 37132 USA; 3 Research Center, BIFIDO Co., Ltd, Hongcheon, 25117 Korea

**Keywords:** Human interleukin-10, *Bifidobacterium bifidum*, Secretion, Bioactive, Recombinant, Expression vector

## Abstract

**Background:**

*Bifidobacterium* spp. are representative probiotics that play an important role in the health of their hosts. Among various *Bifidobacterium* spp*.*, *B. bifidum* BGN4 exhibits relatively high cell adhesion to colonic cells and has been reported to have various in vivo and in vitro bio functionalities (e.g., anti-allergic effect, anti-cancer effect, and modulatory effects on immune cells). Interleukin-10 (IL-10) has emerged as a major suppressor of immune response in macrophages and other antigen presenting cells and plays an essential role in the regulation and resolution of inflammation. In this study, recombinant *B. bifidum* BGN4 [pBESIL10] was developed to deliver human IL-10 effectively to the intestines.

**Results:**

The vector pBESIL10 was constructed by cloning the human IL-10 gene under a *gap* promoter and signal peptide from *Bifidobacterium* spp. into the *E. coli-Bifidobacterium* shuttle vector pBES2. The secreted human IL-10 from *B. bifidum* BGN4 [pBESIL10] was analyzed by sodium dodecyl sulphate-polyacrylamide gel electrophoresis (SDS-PAGE), Western Blotting, and enzyme-linked immunosorbent assay (ELISA). More than 1,473 ± 300 ng/mL (*n* = 4) of human IL-10 was obtained in the cell free culture supernatant of *B. bifidum* BGN4 [pBESIL10]. This productivity is significantly higher than other previously reported human IL-10 level from food grade bacteria. In vitro functional evaluation of the cell free culture supernatant of *B. bifidum* BGN4 [pBESIL10] revealed significantly inhibited interleukin-6 (IL-6) production in lipopolysaccharide (LPS)-induced Raw 264.7 cells (*n* = 6, *p* < 0.0001) and interleukin-8 (IL-8) production in LPS-induced HT-29 cells (*n* = 6, *p* < 0.01) or TNFα-induced HT-29 cells (*n* = 6, *p* < 0.001).

**Conclusion:**

*B. bifidum* BGN4 [pBESIL10] efficiently produces and secretes significant amounts of biologically active human IL-10. The human IL-10 production level in this study is the highest of all human IL-10 production reported to date. Further research should be pursued to evaluate *B. bifidum* BGN4 [pBESIL10] producing IL-10 as a treatment for various inflammation-related diseases, including inflammatory bowel disease, rheumatoid arthritis, allergic asthma, and cancer immunotherapy.

## Introduction

*Bifidobacterium* spp. are Gram-positive, anaerobic, naturally occurring microorganisms that rapidly colonize the large intestine of breastfeeding infants and fermentative bacteria that have gained commercial and scientific interest. They have several health-promoting properties, such as the treatment and prevention of gastrointestinal disorders [1], reduction of lactose intolerance [2], protection against pathogens, enhancement of the immune system [3], and cancer prevention [4–6]. Also, several in vitro or in vivo experimental models have indicated the biosafety [7, 8] and benefits of *Bifidobacterium*, including the enhancement of the cell barrier function [9], intestinal immunomodulatory effects [10], the production of antimicrobial substances [11], and the prevention of obesity and allergy [12].

Among the various *Bifidobacterium* sp., *B. bifidum* BGN4 [13] was chosen as a host in this study because of its proven in vitro and in vivo probiotic functionalities such as (i) high cell adhesion onto colon cells[14]; (ii) modulatory effects on macrophage cells and other immune cells [15]; (iii) anti-allergic responses in mouse models [16]; (iv) its potential for use as a treatment of anti-inflammatory bowel disease [17]; and (v) inhibition of cancer cell line growth [18]. Moreover, *B. bifidum* BGN4 is designated as generally recognized as a safe (GRAS) and certified by the US FDA (GRAS Notice #814) [19], so it is regarded as safe for the expression and delivery of biologically active genes for human use. However, one of the major problems in transforming *Bifidobacterium* is that as a Gram-positive cell, *Bifidobacterium* spp. have thick cell walls which reduce transformation efficiency. To overcome this technical issue, we recently reported optimized electroporation conditions, including overcoming the restriction and modification system through GpC Methyltransferase treatment and cell wall weakening through NaCl [20–22]. Through our newly developed transformation protocol, several genes have been cloned and expressed using *B. bifidum* BGN4 as a host. Specifically, bifidobacterial β-galactosidase [20] was expressed in *B. bifidum* BGN4 and used to reduce lactose in milk and β-glucosidase was expressed and used in the conversion of isoflavone glucosides [23], disaccharides, and ginsenosides [24, 25].

Human IL-10 is a non-covalent homodimer in its biologically active form that dissociates into a stable but inactive monomer at pHs below 5.5 [26]. IL-10 acts as a major suppressor of immune response in macrophages and other antigen presenting cells (APCs) associated with the induction of signal transduction and activation of transcription 3 (STAT3) phosphorylation. IL-10 is known to target diverse cells and act as an anti-inflammatory cytokine. Specifically, IL-10 inhibits the transcription of proinflammatory cytokines and control LPS-induced glucose uptake and glycolysis [27–29]. IL-10 is also known to induce epithelial cell proliferation, promote wound repair, and play a critical role in intestinal homeostasis by its anti-inflammatory activities [30], limit thermogenesis and energy expenditure, and decrease inflammatory responses in adipocytes [31]. IL-10 has the potential to treat cancer and human autoimmune diseases, such as inflammatory bowel disease (IBD) [32, 33] and rheumatoid arthritis [28, 33]. According to a recent report, IL-10 was shown to stimulate the immune response instead of suppressing it [28]. However, secondary side effects could be avoided by using lower doses than those used in systemic treatment (intravenous injection, > 20 μg/kg) of normal people [33, 34].

In previous studies, the mucosal administration of recombinant *L. lactis* secreting IL-10 reduced 50% of colitis induced by the administration of dextran sodium sulfate (DSS) or DiNitro-Benzene Sulfonic-acid (DNBS) in mice [32, 35, 36]. A number of studies using recombinant *L. lactis* secreting IL-10 in IBD have proven that the topical treatment of cytokine IL-10 has clinical benefits in vivo [32, 33, 37]. However, the systemic administration of recombinant bacteria secreting human IL-10 (hIL-10) has not demonstrated significant clinical benefits when compared with placebo groups in chronic disease patients (ClinicalTrials.gov Identifier: NCT00729872)[38], which is due to the low final concentration of recombinant hIL-10 in the gastrointestinal tract [33, 39]. Therefore, an objective of this research was to achieve high level expression of hIL-10 in *B. bifidum* BGN4.

In this study, to maximize the immune-modulatory activity of *B. bifidum* BGN4 as a probiotic cell strain, recombinant *B. bifidum* BGN4 [pBESIL10] was developed to deliver a significant level of human IL-10 directly to the intestines. Human IL-10 was extracellularly expressed in *B. bifidum* BGN4 using a *gap* promoter, a signal peptide from *Bifidobacterium*, and shuttle vector pBES2 by BGN4 [pBESIL10]*.* In addition, the production level and biological activity of the recombinant human IL-10 was evaluated in vitro.

## Materials and methods

### Bacterial strains, culture and bacterial growth experiment

*Escherichia coli* DH5α was routinely cultivated in Luria–Bertani broth (LB) (BD Difco™, Sparks, MD, USA) or LB agar (1% of bacto agar was added) at 37 ℃ overnight, and 100 μg/mL ampicillin was added when necessary [40]. *B. bifidum* BGN4 was routinely grown in de-Mann-Rogosa-Sharpe broth (MRS) (BD Difco™) supplemented with 0.05% (w/v) L-cysteine∙HCl at 37 ℃ overnight under anaerobic conditions, and 3 μg/mL chloramphenicol was added for recombinant BGN4. For the production of human IL-10, recombinant BGN4 was cultured in a 1.2 L jar fermenter in a pH-controlled MRSC (MRS with 0.05% (w/v) L-cysteine∙HCl and 3 ug/mL chloramphenicol added) broth.

To produce pH-sensitive hIL-10, recombinant *B. bifidum* BGN4 [pBESIL10] was cultured overnight in 120 mL of MRSC medium, after which the cells were harvested and resuspended in 1.2 L of fresh MRS broth (pH 6.5). Then, *B. bifidum* BGN4 [pBESIL10] was cultured until reaching pH 6.0, and an ammonia solution was added to maintain the pH at 6.8. The bacteria were inoculated at 10% (v/v), sampled after two hours, and thereafter bacterial suspensions were collected every hour for 10 h. Bacterial cell growth was monitored by reading OD_600nm_ spectrophotometrically using a spectrophotometer (Molecular Devices, San Jose, CA, USA).

### Manipulations of nucleic acids

#### Oligonucleotides

The promoter sequence of the *gap* gene was obtained from the *Bifidobacterium longum* genome database (GenBank AD052817). The signal peptide gene was obtained from *Bifidobacterium bifidum* S17 (GenBank NC_014616.1). The target hIL-10 gene fragments were codon-optimized to *Bifidobacterium bifidum* produced by a commercial supplier (IDT DNA, Coralville, IA, USA)*.* The vector pBES2 was used for the shuttle vector between *E. coli* and *B. bifidum.* The vector pBES2 was constructed from a cryptic plasmid pMG1 from *Bifidobacterium longum* MG1 ligated to pUC19 and possessed ampicillin and chloramphenicol resistance markers [41]. The constructed pBESIL10 was proven to be the right vector by sequence processing conducted by a commercial supplier (Macrogen, Seoul, South Korea). The primers used for the In-Fusion Kit Polymerse Chain Reaction (PCR) (Takara Bio, Tokyo, Japan) were designed with the Takara In-Fusion Cloning Tool.

#### Preparation of nucleic acids

Plasmids were extracted with the Plasmid Purification Mini Kit (Nucleogen, Si-heung, South Korea) using sodium dodecyl sulfate (SDS) cell lysis and ethanol precipitation. In the case of *B. bifidum* transformants, titrated lysozyme diluted to 20 mg/mL with 10 mM tris-HCl added to the bacterial pellet. After the extraction and purification of the pBES2 shuttle vector, the restriction enzymes *Xba*I and *Acc*65I (Bio Labs, Ipswich, MA, USA) were used to linearize it. Agarose gel electrophoresis was used to analyze the DNA molecule via size determination and purify the PCR products via a Nucleogen Gel Extraction Kit. The agarose powder was dissolved in 1 X TAE buffer (40 mM Tris-HCl, 10 mM acetic acid, 1 mM EDTA, pH 8.0). The samples were loaded with 6 X loading star (Dyne Bio, Seongnam, South Korea) and either a Dyne 100 bp or a Dyne 1 kb DNA ladder (Dyne Bio) was used as a size marker. The purified samples were qualified and quantified by broad-range (BR) assay via an Invitrogen Qubit 4 Fluorometer (Thermofisher Scientific) and a Nano spectrophotometer (Nano-400, Allsheng, Hangzhou, China).

#### Polymerse chain reaction

PCR was employed for amplifying the target genes, connecting them as one plasmid, and screening the transformants for cloned inserts [42]. PCR was performed on a SimpliAmp™ Thermal Cycler (Thermofisher Scientific, Waltham, MA, USA). PrimeSTAR GXL DNA Polymerase (Takara Bio) was used for GC-rich gene amplification. The annealing temperature and elongation time were optimized for each primer pair, hIL-10 ORF Forward 5′-AGC CCC GGT CAG GGC-3′, and hIL-10 ORF Reverse 5′-TCA ATT CCT AAT TTT CAT CGT CA-3′. After the gene fragments were prepared, they were connected as one plasmid using the In-Fusion HD Cloning kit (Takara Bio) without DNA ligase. As there were more than two fragments, including the linearized vector, the homologous region of the primer and gene fragment was made to be about 20 bp at the 5′ end of the particular DNA strand. The PCR conditions were determined by the primer pairs: for *Pgap*, Pgap Forward, 5′-TGA TAA TAA GGG TAC TCT AGA GAT CTG GGG AAT GCC TCG-3′, and Pgap Reverse, 5′-GAC CGG GGC TAT CCG CTG CGT TGG CCG TG-3′; for *hIL-10 ORF*, hIL-10 ORF Forward, 5′-CCA CGG CCA ACG CAG CGG ATA GCC CCG GTC AGG GCA-3′ and hIL-10 ORF Reverse, 5′-GGT ACC CGG GGA TCC TCT AGT AAT GCC AAC TTT GTA CAA GAA AGC-3′. After PCR cycling was completed, chemo-transformation was conducted on *E. coli* DH5 α (Transgen Biotech, Beijing, China). The PCR was also used to screen the transformants from among other bacteria with 2× EmeralAmp GTPCR Master Mix (Takara Bio). The primers, Ligation Forward 5′-TTA AAT ATC TCT TTT CTC-3′ and Ligation Reverse 5′-TCA ATT CCT AAT TTT CAT CGT CA-3′, were used to screen whether the plasmid was properly constructed.

#### Construction of pBESIL10

*Bifidobacterium-E. coli* shuttle vector pBES2 was constructed using a cryptic plasmid from *B. longum* MG1 ligated with pUC19 to possess ampicillin and chloramphenicol resistance markers [41]. pBES2 was cut by *Xba*I and *Acc*65I. The promoter with the signal peptide and hIL10 ORF with a terminator were amplified via PCR and connected with vector pBES2 via In-fusion cloning PCR.

#### Plasmid transformation to Bifidobacteria

Plasmid DNA extracted from *E. coli* DH5α was methylated by GpC (M.CviPI) methyltransferases (NEB, Ipswich, MA, USA) and purified using a Nucleogen Gel Extraction Kit. After the pBESIL10 was constructed via Infusion PCR, restriction enzymes (Xba1, Acc65I) were used to confirm whether the plasmid was correctly transformed into *E. coli* DH5α. Electrocompetent *B. bifidum* BGN4 was prepared and transformed according to the method of Park et al. [21, 22]. After culturing the recombinant bacteria on a MRSC agar plate for 36 h, the colonies formed on the selective plates were extracted and sequenced with primers from a commercial supplier (Macrogen) to determine whether they were the correct transformants.

### Expression of human interleukin-10

#### Protein production and separation

For the production of hIL-10, BGN4 [pBESIL10] was cultured in MRS broth in a 1.2 L jar fermenter at pH 6.8. As a control group, BGN4 [pBES2] without hIL-10 was cultured under the same conditions. The bacteria were inoculated at 10% (v/v), sampled after two hours, and thereafter bacterial suspensions were collected every hour for 10 h. After measuring the OD_600nm_ value, the sampled bacterial suspensions were immediately centrifuged (13,000 rpm, 10 min).

Five options to precipitate the protein from the cell free culture supernatant (CFCS) were evaluated (TCA, acetone, ethanol, TCA ethanol, amicon precipitation, and chloroform methanol); chloroform methanol precipitation was chosen for further experiments because the other methods did not produce detectable IL-10. The protein was extracted from CFCS with 4 volumes of methanol, 1 volume of chloroform, and 3 volumes of distilled water, and centrifuged (14,000*g*, 1 min). After centrifugation, the aqueous layer of the mixture was removed, 4 volumes of methanol was added, and it was centrifuged again (14,000*g*, 2 min). MeOH was removed via speed-vacuum (Labo Gene, Copenhagen, Denmark) and the residual was dissolved in a RIPA buffer (Elpis Biotech, Daejeon, South Korea).

To detect intracellular hIL-10, the cell pellets were resuspended in TNT buffer (50 mM Tris-HCl, pH 8, 300 mM NaCl, 0.1% Triton X-100) and treated with 5 mg/mL lysozyme diluted in 20 mM Tris–HCl for one hour at 37 ℃ [43, 44]. The resuspended cell pellets were centrifuged (10,000*g*, 3 min) and washed three times. The washed cell pellets were resuspended in TNT buffer and sonicated (5 min, 1–0-1–0 pulse). The sonicated solutions were centrifuged (10,000 g, 10 min) and dissolved in RIPA buffer (Elpis Biotech).

#### ELISA analysis

A sandwich enzyme-linked immunosorbent assay (ELISA) was used to assess the amount of human intrleukin-10 (hIL-10), mouse IL-6, and human IL-8 secreted in the CFCS of recombinant bacteria, the HT-29 cell, and Raw 264.7 cell supernatants. The routinely grown BGN4 [pBESIL10] and BGN4 [pBES2] in MRSC at 37 ℃ overnight (18 h) under anaerobic conditions of 120 mL culture broth were harvested by centrifugation. Then, all of the pellets were inoculated into individual 1.2 L of fresh MRS broth (pH 6.5) at 10% (v/v) at 37 ℃. The bacterial supernatants were collected every hour and analyzed with a BD OptEIA™ kit (BD Biosciences, San Jose, CA, USA). The cell supernatants were collected after 6 h or 4 h treatment. Anti-human IL-10, anti-mouse IL-6, and anti-human IL-8 monoclonal antibodies were used as capture antibodies. The cell supernatants were collected after being treated by the bacterial supernatants [45].

#### Western blot analysis

For protein quantification in extracts from the CFCS and cell pellets of the recombinant bacteria, Bradford Assays (Bio-Rad) were used. Total protein concentration was calculated by a standard curve using several dilutions of BSA standard solution. Western Blot analysis was conducted to confirm the expression of hIL-10 in *B. bifidum* BGN4 [pBESIL10]. The protein fractions from the CFCS and cell pellets were separated by SDS-PAGE and blotted onto a 15% PVDF membrane (Bio-Rad). A precision plus protein dual color standard (Bio-Rad) was used as a molecular weight ladder, and a recombinant human IL-10 protein (LS-Bio, Seattle, WA, USA) was used as a positive control. A human IL-10 monoclonal antibody (JES3-12G8, Thermofisher Scientific) diluted to 1:100 and Pierce Goat Anti-Rat IgG (H + L) peroxidase conjugated secondary antibody (31470, Thermofisher Scientific) diluted to 1:500 were used. Specific bands on the membrane were visualized with an enhanced chemiluminescence (ECL) solution from the Western Bright ECL Kit (Advansta, San Jose, CA, USA).

### Bioassay

#### Cell line, media, and culture condition

Raw 264.7 KCLB 40071) and HT-29 (KCLB 30038) cell lines were purchased from the Korea Cell Line Bank (Seoul, South Korea). Raw 264.7 cells (10^6^ cells/well), and HT-29 cells (10^6^ cells/well) were seeded into 24-well plates in Dulbecco’s Modified Eagle’s Medium (DMEM, Sigma-Aldrich) with 10% heat-inactivated fetal bovine serum (FBS, Welgene Inc., Daegu, South Korea) and 1% antibiotic antimycotic solution (Sigma-Aldrich) and cultured for two to three days at 37 ℃ in an atmosphere of 5% CO_2_ /95% air.

#### MTT cell viability assay

The mitochondria-dependent reduction of MTT (3-[4,5-dimethylthiazol-2-yl]-2,5 diphenyl-2H-tetrazolium bromide) to formazan was performed to evaluate the effect of CFCS on the viability of the cells. Raw 264.7 cells were prepared at 100 μL of 10^6^ cells/mL for each well in 96-well plates at 37 ℃ in an atmosphere of 5% CO_2_/95% air for 24 h. Thereafter, the cells were treated with CFCS with and without 1 μg/mL LPS for 24 h. After removing the cell culture supernatant solutions, 100 μL of a diluted MTT solution (5 mg/mL of MTT solution in PBS buffer diluted in DMEM 1:10) was added to each well and they were incubated for three hours. Then, 50 μL of dimethyl sulfoxide (DMSO) was added to each well to solubilize the formazan. The optical density was measured at 570 nm via a microplate reader (Molecular Devices).

#### Evaluation of anti-inflammatory effects of *B. bifidum* BGN4 [pBESIL10]

The cultured cells were stimulated with 100 ng/mL or 1 μg/mL of lipopolysaccharide (LPS) (Sigma Aldrich) or 0.5 ng/mL of TNFα (PeproTech, Hamada, Israel). The cells were treated with 5% or 10% (v/v) CFCS of recombinant bacteria BGN4 [pBES2] and BGN4 [pBESIL10] or a recombinant hIL-10 protein in a total volume of 500 μL for 6 h or 4 h of treatment. All samples were analyzed in triplicate. After incubation, cell culture supernatants were collected and stored at – 80℃ until further analysis. The active recombinant human IL-10 protein was used as a control group. The effect of ten percent (v/v) of hIL-10 secreted by the BGN4 [pBESIL10] 10 h cultured group was similar to that of 150 ng/mL of rIL-10.

The presence of nitrite was determined in the cell culture media via a NO detection assay. One hundred μL of cell culture medium with an equal volume of Griess reagent (Thermofisher Scientific) in a 96-well plate was incubated at room temperature for 10 min. Then, the absorbance was measured at 540 nm using a microplate reader (Molecular Devices). The amount of nitrite in the supernatant was calculated from a sodium nitrite (NaNO_2_) standard curve. Also, the cell culture media were collected to determine the suppression of mIL-6 and hIL-8 by mIL-6 and hIL-8 ELISA kits (BD Biosciences).

#### Statistical analysis

The values of hIL-10 (ng/mL) in the CFCS are the means of the replicate determinations ± standard deviation. The other values are the means of the replicate determinations + standard deviation. The ELISA data was analyzed by the GraphPad Prism (Ver 8.0.1) using an ordinary one-way ANOVA test. The statistical differences were examined by Tukey’s multiple comparison tests. The statistical significance was *p* < 0.05.

## Results

### Construction of pBESIL10

The promoter regions upstream of the *gap* gene *(bbif_0612)* encoding glyceraldehyde-3-phosphate dehydrogenase (P*gap*) were predicted using the BPROM online software tool (http://linuxl.softberry.com/BPROM), which was developed for bacterial promoter prediction based on the recognition motif of *E. coli* σ^70^ type sigma factors. This analysis yielded a single promoter with a relatively high linear discrimination function (LDF) in front of *gap* when the default unchangeable threshold value equals 0.2.

According to prior research, the transcriptional activities of four different putative promoters have been assayed by β-glucuronidase activity [46]. The transcriptional activity of P_*gap*_ was found to be significantly higher than that of the other promoters (P_*hup*_, P_*huxS*_, P_*16S,*_* P*_*rRNA*_) of *B. bifidum*. Since the promoter region of the *gap* gene of *B. bifidum* S17 yielded the highest transcriptional activity among the promoters tested, P_*gap*_ was used as a promoter of the plasmid in this study (data not shown).

According to prior research, the functionalities of various signal peptides from three bifidobacterial strains, *B. bifidum* S17, *B. longum* E18, and *B. breve* S27, have been tested based on phytase activities in the supernatants of *B. bifidum* S17 constructs [46]. Since the S6 signal peptide (*bbif_1671)* was the most effective protein secretion signal from *B. bifidum* S17, it was chosen as a signal peptide in this study (data not shown). The DNA sequences of the signal peptide S6 (*bbif_1671)* are:

ATGAAATCACTGATGAAAAAGGTTTTCGCTGCCGCCGCGGCGATTGCCA

CCGTATTTGGATTGGCTGCGACGACAGTCGCCACGGCCAACGCAGCGGAT

The amino acid sequence of the signal peptide S6 (*bbif_1671)* is:

MKSLMKKVFAAAAAIATVFGLAATTVATANA*AD

(The * indicates predicted cleavage sites)

**Fig. 1:**
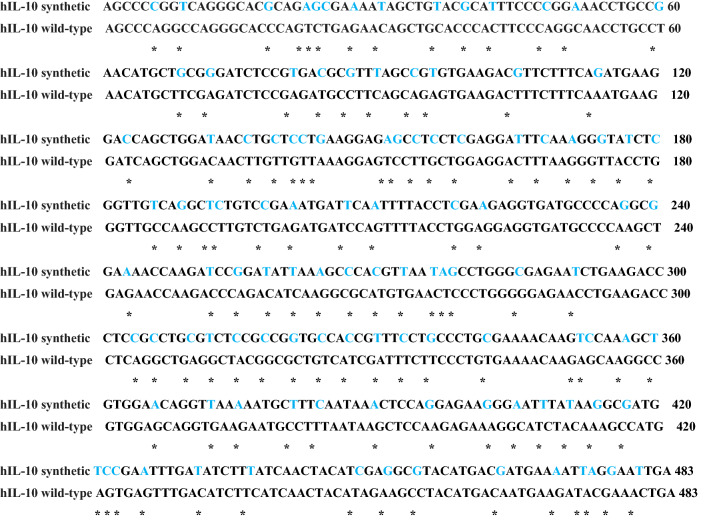
Alignment of nucleotide sequences of the original and codon-optimized hIL-10 gene (* is the modified sequence from the original hIL-10 gene during codon-optimization)

The synthetic hIL-10 gene was designed by the preferred codons to be expressed in *B. bifidum*. The optimized synthetic hIL-10 gene is shown in Table 1. The optimized gene had 79% identity with the original gene (Fig. 1).

**Table 1:**
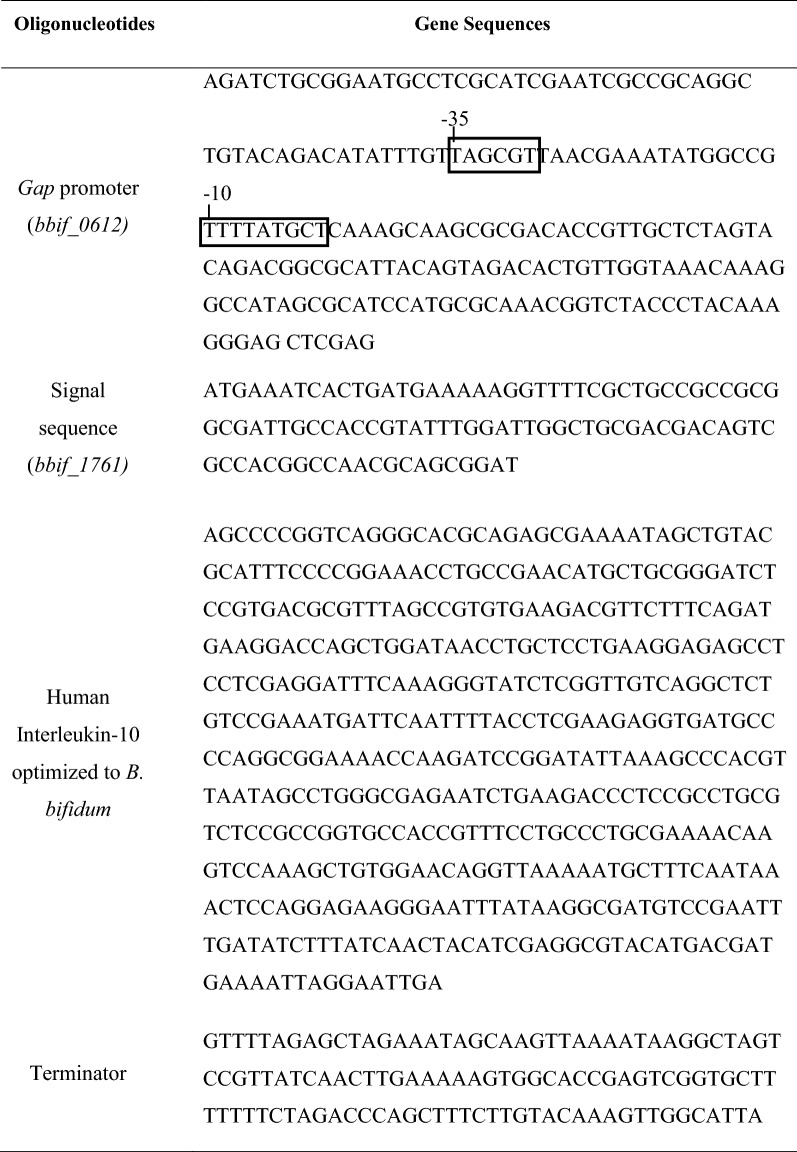
Components of pBESIL10

### Confirmation of BGN4 [pBESIL10] and growth curve

**Fig. 2:**
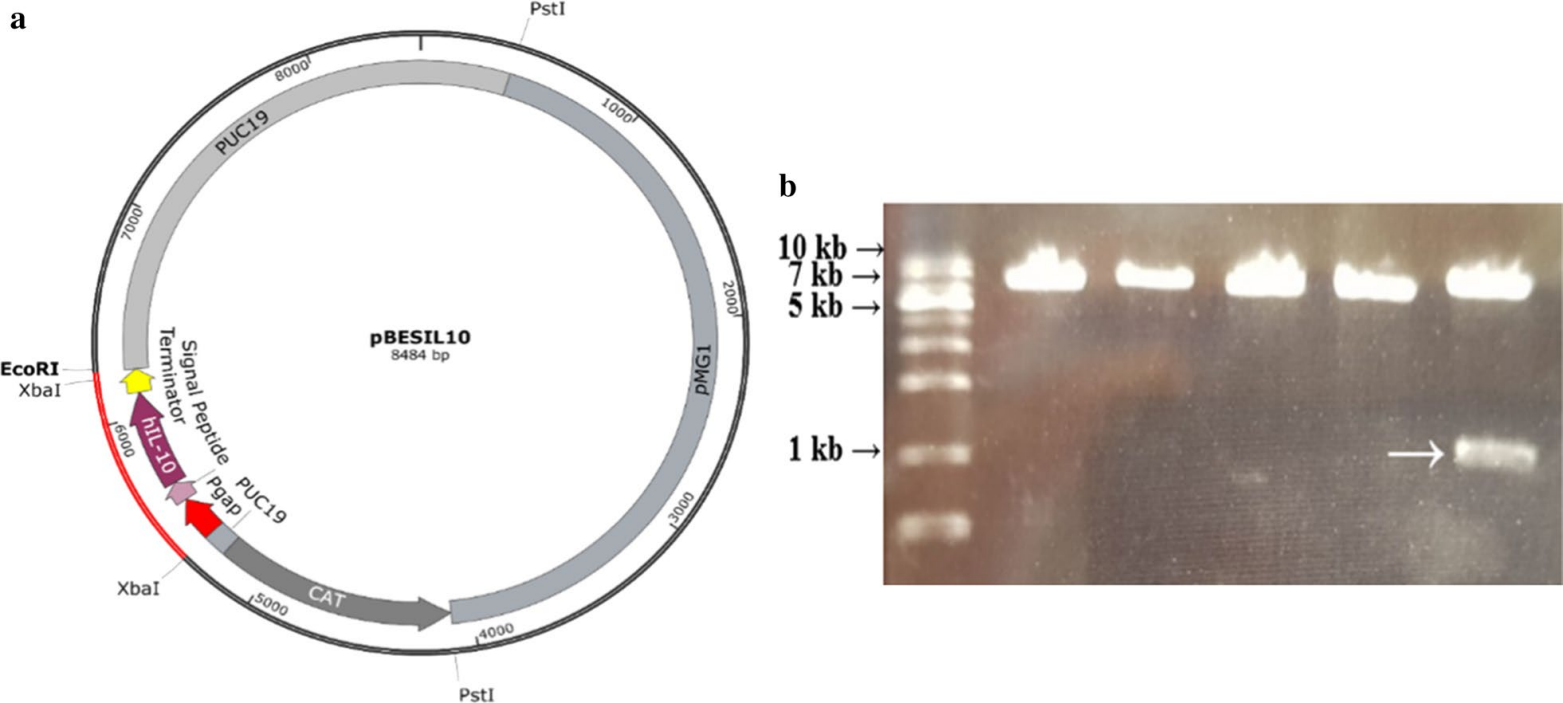
**a** The structure of pBESIL10. **b** Restriction assay of pBES2 and pBESIL10. pBESIL10 is constructed with P*gap* (*gap* promoter), the signal peptide of gene *bbif_1761,* hIL-10 (human Interleukin-10 ORF gene) optimized for *Bifidobacterium bifidum*, and terminator into pBES2. *CmR* and *AmpR* are chloramphenicol and ampicillin resistance genes; *Ori* is the origin of replication from pUC19*; Mob* and *Rep* are plasmid–encoded protein of MG1 from *Bifidobacterium longum* MG1 [38]*.* **b** Lane 1: 1 kb DNA ladder; Lane 2, 3, 4, 5: Xba1–Acc65I restriction of pBES2 shows one fragment (7.5 kb), as the other fragment is too small; Lane 6: Xba1–Acc65I restriction of pBESIL10 shows a 0.9 kb fragment (Insert fragment with Pgap, signal peptide, hIL-10 (optimized) and terminator connected, white arrow) with cut pBES2 (7.5 kb)

pBESIL10 was constructed as discussed in the Materials and Method section, and the map is presented in Fig. 2a and Table 1. The size of the pBESIL10 is 8484 bp and the organized plasmid secretes hIL-10 protein in the recombinant *B. bifidum* BGN4 [pBESIL10]. After the pBESIL10 was constructed via Infusion PCR, restriction enzymes (Xba1, Acc65I) were used to confirm whether the plasmid was correctly transformed into *E. coli* DH5α (Fig. 2b). pBES2 digested with restricted enzymes was revealed as a fragment similar in size to pBES2 (7.5 kb), as the cut fragment was too small. However, pBESIL10 digested with restricted enzymes appeared as two fragments: a 0.9 kb insert fragment and a 7.5 kb larger fragment.

**Fig. 3:**
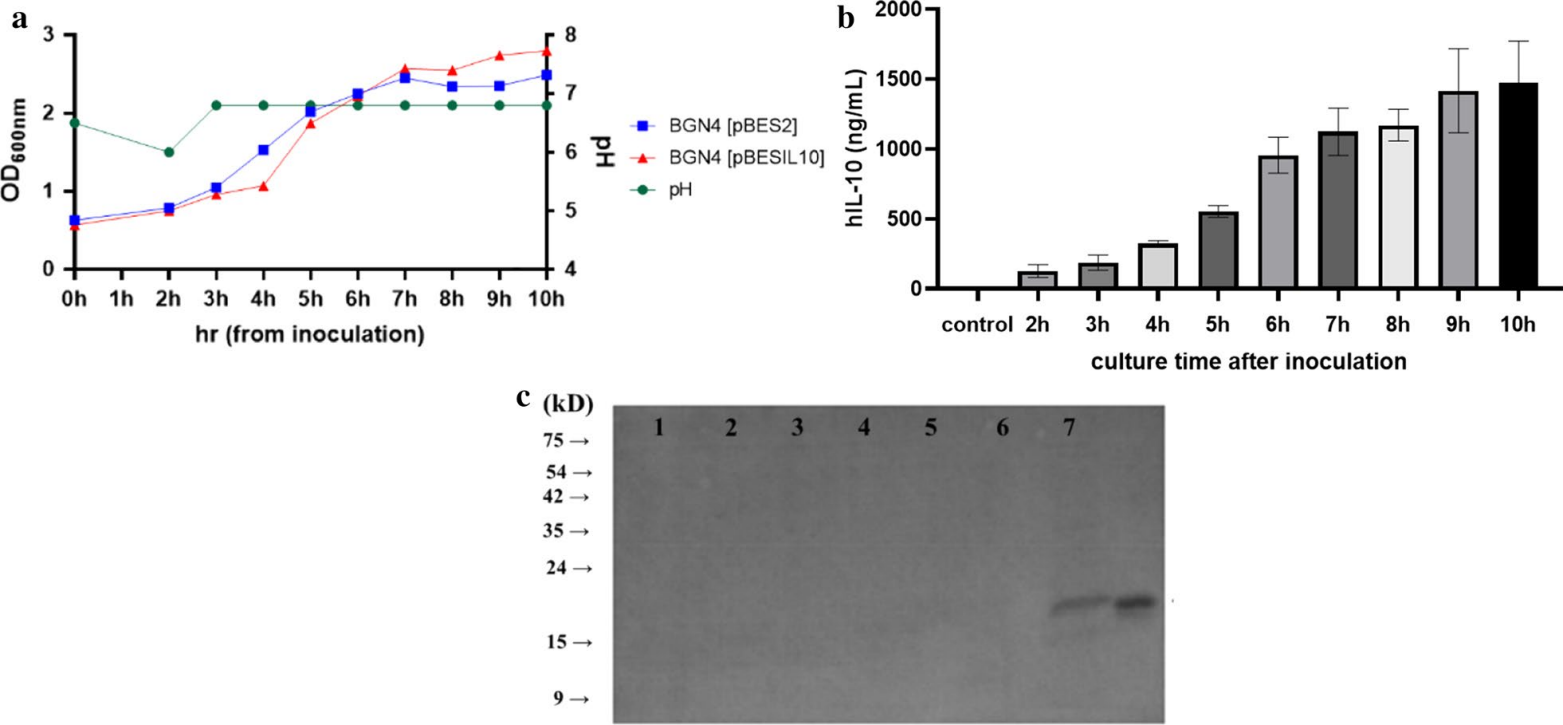
Bacterial growth and hIL-10 production over time. **a** Culturing recombinant BGN4 graph at OD_600nm_. **b** Quantification of hIL-10 produced by BGN4 [pBESIL10] Cell Free Culture Supernatant (CFCS) during culture in pH-controlled MRS medium by sandwich ELISA. (c) Western blot detection of human IL-10 protein. **a** The 120 mL of cultured recombinant bacteria were centrifuged and inoculated into 1.2 L of a fresh MRS broth (pH 6.5) at 0 h each. BGN4 cells were cultured until reaching a pH of 6.0, and a diluted ammonia solution was added to maintain a pH of 6.8 during the bacteria culture. **b** The pH was maintained at 6.8 during cultivation and the negative control BGN4 [pBES2] CFCS did not show detectable hIL-10 at the same condition. All data are shown as the mean ± standard deviation (*n* = 4). The amount of hIL-10 were: (2 h) 128 ± 46 ng/mL, (3 h) 189 ± 54 ng/mL, (4 h) 327 ± 20 ng/mL, (5 h) 554 ± 42 ng/mL, (6 h) 956 ± 129 ng/mL, (7 h) 1,124 ± 170 ng/mL, (8 h) 1,171 ± 114 ng/mL, (9 h) 1,416 ± 300 ng/mL and (10 h) 1,473 ± 300 ng/mL, respectively. (c) All CFCS were concentrated 40-fold by different precipitation methods. Lanes 1, 3, 5: BGN4 [pBES2] control strain grown in buffered MRS medium (pH 6.8); Lanes 2, 4, 6, 7: BGN4 [pBESIL10] strain grown in buffered MRS medium (pH 6.8); Lane C: positive control (recombinant hIL-10 protein (1 μg)); Lanes 1, 2: ethanol precipitation; Lanes 3, 4: acetone precipitation; Lanes 5, 7: chlorophenol methanol precipitation; Lane 6: TCA precipitation (other supernatant precipitation data and cell pellet data are not shown)

BGN4 [pBESIL10] and BGN4 [pBES2] were collected every hour. The time that the bacteria were inoculated was 0 h in Fig. 3a. The recombinant bacteria BGN4 [pBESIL10] growth curve appeared similar to that of BGN4 [pBES2] (Fig. 3a).

### Expression of recombinant hIL-10

#### ELISA

The production of hIL-10 in the CFCS of recombinant *B. bifidum* BGN4 was measured by a BD ELISA kit according to the growth of recombinant BGN4 (Fig. 3a). The *B. bifidum* BGN4 [pBES2] cultured in buffered MRS for 10 h group was used as a negative control (Fig. 3b). *B*. *bifidum* BGN4 [pBESIL10] showed growth-associated production of hIL-10 (*n* = 4). The first bacteria supernatant (2 h) contained 128 ± 46 ng/mL of hIL-10, the fifth bacteria supernatant (6 h) contained 956 ± 129 ng/mL, and the last bacteria supernatant (10 h) contained 1473 ± 300 ng/mL of hIL-10. Thus the 6 h to 10 h groups secreted a significantly larger amount of hIL-10. The 9 h and 10 h groups secreted significantly much more hIL-10 when compared with the 6 h group. During the 10 h of cultivation, the negative control showed hIL-10 concentrations below the detection limit.

#### Western blot analysis

The largest secretory production of hIL-10 in the CFCS of the BGN4 [pBESIL10] 10 h group was confirmed by Western Blot. The CFCS of bacteria cultured for 10 h in buffered MRS was concentrated 40-fold by six different protein precipitation methods using TCA, acetone, ethanol, TCA ethanol, amicon precipitation, and chloroform methanol. Only the protocol using chloroform methanol showed the expected protein precipitation from the CFCS of BGN4 [pBESIL10]. The protein concentration of the extracted samples was quantified with a Bradford assay. According to the Bradford standard curve, the loading sample volumes were determined by 20 μg of the total proteins. For low-concentration samples, 15 μL of the samples were loaded depending on the maximum loading capacity. The CFCS of BGN4 [pBESIL10] precipitated with chlorophenol methanol was the only supernatant that exhibited the hIL-10 protein in the Western Blot (Fig. 3c).

### Bioassay

#### MTT assay–cell viability in raw 264.7 cells

**Fig. 4:**
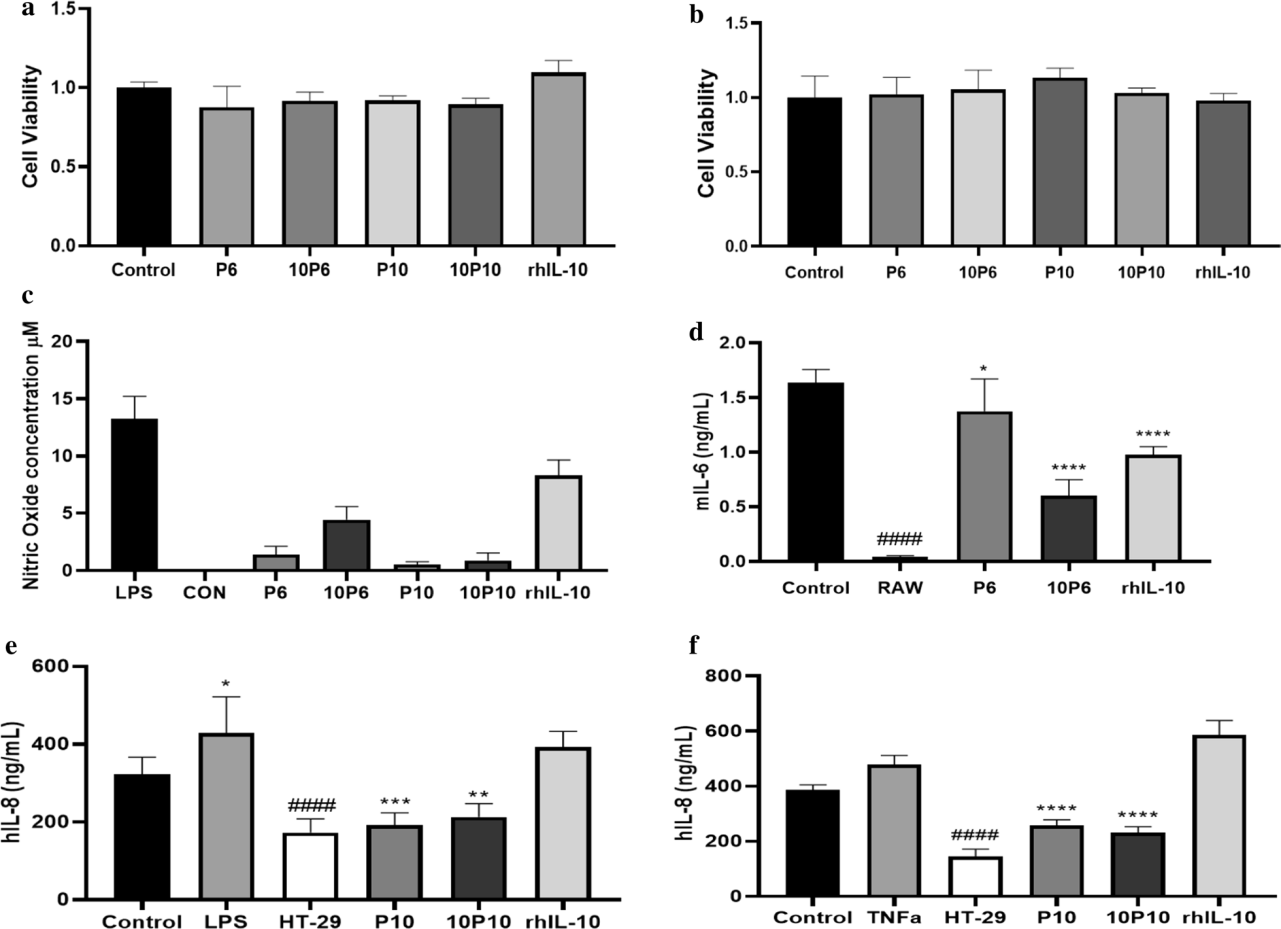
Bioassay with Raw 264.7 cells and HT-29 cells treated with 10% (v/v) cell free culture supernatant (CFCS) of BGN4 [pBES2] and BGN4 [pBESIL10] or rhIL-10 protein. rhIL-10 protein is an active recombinant human interleukin-10 protein, 150 ng/mL of which was similar to the 10% (v/v) of hIL-10 secreted by the BGN4[pBESIL10] 10 h cultured group. Viability of Raw 264.7 cells cultured in DMEM media for 24 h without LPS (**a**) and with 100 ng/mL of LPS (**b**). The inhibition of nitric oxide (NO) (**c**). The effects of CFCS of BGN4 [pBESIL10] on IL-6 production in (d) LPS-stimulated RAW 264.7 macrophage cells, IL-8 production in LPS- (**e**) or TNFα- (**f**) stimulated on HT-29 cells. Each data point represents the mean + standard deviation (*n* = 6). **a**, **b** Raw 264.7 cells (10^5^/well) were treated with 10% (v/v) CFCS of BGN4 [pBES2], BGN4 [pBESIL10] or rhIL-10 protein. Control: 10% (v/v) MRS; P6, P10: CFCS of BGN4 [pBES2] prepared at 6 h or 10 h of cultivation in buffered MRS; 10P6, 10P10: CFCS of BGN4 [pBESIL10] prepared at 6 h or 10 h of cultivation in buffered MRS; rhIL-10: 1 μg/mL of recombinant hIL-10 protein. There was no statistically significant difference among the treatment groups in both graphs with *p* < 0.05. **c**, **d** Raw 264.7 cells (5 × 10^5^ /well) were cultured in DMEM media for 6 h with 100 ng/mL of LPS and 10% (v/v) CFCS of bacteria. **c** LPS: LPS 100 ng/mL treated only; P6, P10: CFCS of BGN4 [pBES2] prepared at 6 h or 10 h of cultivation in buffered MRS with LPS 100 ng/mL; 10P6, 10P10: CFCS of BGN4 [pBESIL10] prepared at 6 h or 10 h of cultivation in buffered MRS with LPS 100 ng/mL; rhIL-10: 1 μg/mL of recombinant hIL-10 protein with LPS 100 ng/mL; CON: DMEM media only. Untreated control group exposure data was below the detection limit. For all samples: *p* < 0.0001 (against LPS-stimulated group). **d** Control: 10% (v/v) MRS; RAW: Raw 264.7 cells without any treatment; P6: CFCS of BGN4 [pBES2] 6 h cultivation in buffered MRS; 10P6: CFCS of BGN4 [pBESIL10] 6 h cultivation in buffered MRS; rhIL-10: 0.1 μg/mL of recombinant hIL-10 protein. **e**, **f** HT-29 cells (5 × 10^5^/well) were incubated in DEMEM culture media for 4 h with 5% (v/v) CFCS of bacteria. Control: 5% (v/v) MRS; HT-29: HT-29 cells without any treatment; P10: CFCS of BGN4 [pBES2] 10 h cultivation in buffered MRS; 10P10: CFCS of BGN4 [pBESIL10] 10 h cultivation in buffered MRS; rhIL-10: recombinant hIL-10 protein 0.1 μg/mL; LPS: LPS 1 μg/mL; TNFα: TNFα 0.5 ng/mL. ^####^*p* < 0.0001, the control group versus the untreated group; *p < 0.1, **p < 0.01, ***p < 0.001, ****p < 0.0001, the treated group was significantly different from the control group

An MTT assay was used to analyze the effects of the CFCS of BGN4 [pBES2] and BGN4 [pBESIL10] cultured in buffered MRS media (Fig. 3b) as well as the recombinant protein hIL-10 (rhIL-10) on Raw 264.7 cell viability. As the results in Fig. 4 show, hIL-10 production increased with time, especially for the 6 h to 10 h groups. Therefore, the cells were treated by the 6 h or 10 h group CFCS of BGN4 [pBES2] and BGN4 [pBESIL10], respectively. The Raw 264.7 cells (10^5^ cells/well) were treated by various CFCS with/without LPS for 24 h (Fig. 4a, b).

Figure 4a shows the cell viability of Raw 264.7 cells without LPS treatment. All cell viabilities treated with 10% (v/v) of each CFCS were over 85% against the control (only MRS media 10% (v/v) treated) (*n* = 6). Figure 4b shows the cell viability of Raw 264.7 cells treated by each CFCS with 100 ng/mL of LPS. The cell viabilities treated with the samples and LPS were over 95% against the control (LPS 100 ng/mL with MRS 10% (v/v)) (*n* = 6). MTT assay results showed no obvious cytotoxicity of CFCS of BGN4 [pBES2] and BGN4 [pBESIL10] or rhIL-10 after 24 h incubation in both the LPS-treated and LPS-untreated mouse macrophage.

#### Inhibition of nitric oxide (NO) production in LPS-stimulated raw 264.7 cells

Nitrite accumulation in the cells increased due to LPS treatment. Nitrite production was measured in the culture medium of the Raw 264.7 (5 × 10^5^ /well) murine macrophage cell line. Each of the cells were treated with the CFCS of *B. bifidum* BGN4 [pBES2] and *B. bifidum* BGN4 [pBESIL10] and recombinant protein hIL-10 (Fig. 4c). The Raw 264.7 cells were activated by 100 ng/mL of LPS, and NO production was measured as the nitrite concentration in the cell culture medium. The untreated control group exhibited nitrite concentration below the detection limit. Comparing the LPS group and the sample groups treated with LPS, the sample-treated groups released a significantly lower level of NO in the media (*n* = 6, *p* < 0.0001). The CFCS of the recombinant bacteria showed a significantly higher reduction of nitrite accumulation in the LPS-stimulated Raw 264.7 cells.

#### Inhibitory effect on the expression of pro-inflammatory cytokine IL-6 in LPS-stimulated raw 264.7 cells

IL-6 is one of the pro-inflammatory cytokines that participates in the prolongation of chronic inflammation. The level of the cytokine production in LPS-induced macrophages was evaluated using ELISA. Raw 264.7 cells (5 × 10^5^ /well) treated with LPS 100 ng/mL for 6 h had significantly increased IL-6 production (Fig. 4d) compared to the untreated Raw 264.7 cells. The CFCS of BGN4 [pBESIL10] significantly suppressed the production of cytokine IL-6. The cells stimulated with LPS treated with the CFCS of BGN4 [pBESIL10] 6 h cultured group in buffered MRS and 10 h cultured group in buffered MRS (data not shown) for 6 h significantly suppressed the production of cytokine IL-6. BGN4 [pBESIL10] (*n* = 6, *p* < 0.0001) was more effective in suppressing IL-6 production than BGN4 [pBES2] (*n* = 6, *p* < 0.1) and 0.1 μg/mL of the recombinant hIL-10 protein (*n* = 6, *p* < 0.0001).

#### Inhibitory effect on the expression of pro-inflammatory cytokine IL-8 in LPS- or TNFα–simulated HT-29 cells

IL-8 is one of the pro-inflammatory cytokines that participates in the prolongation of chronic inflammation. Cytokine production in LPS- and TNFα-induced cells was evaluated using ELISA. The HT-29 cells (5 × 10^5^ /well) treated with 1 μg/mL LPS or 0.5 ng/mL TNFα for 4 h showed a significant increase in IL-8 production when compared to the untreated HT-29 cells. Figure 4e shows the CFCS of BGN4 [pBESIL10] 10 h cultured group (*n* = 6, *p* < 0.01) in buffered MRS and BGN4 [pBES2] 10 h cultured group (*n* = 6, *p* < 0.001) in buffered MRS significantly suppressed the production of cytokine IL-8. Figure 4f shows the CFCS of the BGN4 [pBESIL10] 10 h cultured group (*n* = 6, *p* < 0.0001) in buffered MRS and BGN4 [pBES2] 10 h cultured group (*n* = 6, *p* < 0.0001) in buffered MRS significantly suppressed the production of cytokine IL-8. Both were present at levels similar to the untreated HT-29 cells. However, 0.1 μg/mL of the recombinant hIL-10 protein did not suppress the production of cytokine IL-8.

### Comparing secretion of IL-10 with other recombinant probiotics

Several previous studies involving genetically modified probiotics secreting IL-10 are shown in Table 2. Some secreted murine IL-10, which is not available in human studies, as the human IL-10 receptor does not bind with murine IL-10 [26, 29]. Specifically, *L. lactis* [pLB263] [47], *L. lactis* MG1363 [pT1MIL10] [44], *B. bifidum* BS42 [pBEST_SP1181_:IL-10] [48], and *B. bifidum* BS42 [pBEST_SP1181_:IL-10] [48] secreted murine IL-10.

**Table 2:** Various recombinant probiotics secreting IL-10

Probiotics	Protein	Vector	Promoter	amount	References
*L. lactis Thy12*	Human IL-10	Chromosome integrated	P_thyA_ (constitutive)	13.7 ng/mL	[35]
*L. lactis*	Murine IL-10	pLB263	P_groESL_ (Inducible)	40.72 ng/mL	[45]
*L. lactis* MG1363	Murine IL-10	pT1MIL10	P1 (constitutive)	630 ng/mL	[41]
*B. longum* NCC2705	Human IL-10	pLR	P_hup_ (constitutive)	22 pg/mL	[40]
*B. breve* UCC2003	Human IL-10	pESH100	P_gap_ (constitutive)	1.9 ng/mL	[47]
*B. bifidum* BS42	Murine IL-10	pBEST_Exp4_:IL-10	P_dnaK_ (constitutive)	7 ng/mL	[46]
*B. bifidum* BS42	Murine Il-10	pBEST_SP1181_:IL-10	P_dnaK_ (constitutive)	24 ng/mL	[46]
*B. bifidum* BGN4	Human IL-10	pBESIL10	P_gap_ (constitutive)	1473 ng/mL	This study

Also, there are some *Bifidobacteria* secreting IL-10, however, the amount is much lower that of BGN4 [pBESIL10]. For instance, a recombinant strain of *B. longum* ATCC 1507 [pLR] has been reported to produce 22 pg/mL of IL-10 [43]; *B. breve* [pESH100] produced 1900 pg/mL of hIL-10 [49]; *B. bifidum* BS42 [pBEST_Exp4_:IL-10] produced 1000 pg/mL of mIL-10; *B. bifidum* BS42 [pBEST_SP1181_:IL-10] produced 7000 pg/mL of mIL-10; and, at pH 8, *B. bifidum* BS42 [pBEST_SP1181_:IL-10] overproduced mIL-10 24,000 pg/mL [48].

In our study, *B. bifidum* BGN4 [pBESIL10] produced the greatest amount of hIL-10 compared to those described with other plasmids with transformed probiotics, with a yield of 1,473 ± 300 ng/mL of hIL-10.

## Discussion

IL-10 has been well-documented as a major suppressor of the inflammatory immune response in diverse cells, and has been proven by various in vitro and in vivo studies [29, 30, 50]. There have been a number of studies using recombinant *L. lactis* and other *Bifidobacterium*-secreting IL-10 related to IBD treatment and clinic benefits [35, 43, 44, 46]. For example, recombinant *Lactococcus lactis* secreting hIL-10 in Crohn’s disease was successful in phase 1 clinical trial. As the secretion of IL-10 by recombinant probiotic bacteria can be delivered directly to the intestine, the risk of degradation is small, and it is easy to increase the concentrations of IL-10 in the intestine [33]. In this study, human IL-10 was cloned and transformed into *Bifidobacterium bifidum* BGN4, and its successful expression and activity were proven by ELISA, Western Blot, and bioassay testing.

Macrophages and lymphocytes play a central role in immune response through the release of different cytokines, and the evaluation of the levels of cytokines and macrophage activity using an in vitro assay is considered an indirect way to analyze bio-functional effects of recombinant bacteria [51]. Through the MTT test of RAW 264.7 cells with and without LPS treatment, cell viability was determined not to be affected by the cell free culture supernatant (CFCS) of *B. bifidum* BGN4 [pBES2] and *B. bifidum* BGN4 [pBESIL10]. RAW 264.7 cells activated by LPS treatment produce nitric oxide (NO), which is an important mediator of macrophage phagocytosis [52]. The CFCS of BGN4 induced well-developed morphological changes in the macrophages and increased phagocytic activity, thus significantly reducing NO production [15]. Also, IL-10 limits basic microbicidal mechanisms, such as the production of NO in murine macrophages [53]. Therefore, *B. bifidum* BGN4 [pBES2] and *B. bifidum* BGN4 [pBESIL10] supernatants both significantly lower NO levels.

The recombinant *B. bifidum* BGN4 [pBESIL10] secreting human IL-10 also significantly lowered the inflammatory index (IL-6) in mouse RAW 264.7 cells. IL-6, which is a pro-inflammatory cytokine, has immunomodulatory effects, including the stimulation of B-cell differentiation and T-cell activation [54]. As the *B. bifidum* BGN4 supernatant was unable to affect IL-6 production [15, 55], the *B. bifidum* BGN4 [pBES2] supernatant could not reduce the IL-6 production. As IL-10 could significantly inhibit IL-6 protein production at high concentrations of LPS treatment in macrophage cell lines [56], human IL-10 secreted by the *B. bifidum* BGN4 [pBESIL10] supernatant significantly inhibited IL-6 production in RAW 264.7 cells.

*Bifidobacterium* sp. inhibits IL-8 release in LPS-treated human colon adenocarcinoma (HT-29) cells, and these *Bifidobacterium* cell species may be good agents for preventing inflammation via neutralizing Gram-negative endotoxins and improving intestinal health [10, 57]. IL-8, which is one of the proinflammatory cytokines produced in the intestine, initiates an acute inflammatory cascade and is an early marker of the inflammatory process [54]. IL-10 does not stimulate IL-8 generation and has no effect on IL-8 production by LPS or TNFα induced the colonic epithelial cell line HT-29 [58]. The transcriptional control of IL-8 was mediated by transcription factor NF-кB in HT-29 cells, which is up regulated by TNFα [59]. However, hIL-10 affects both Th1 and Th2 and inhibits IL-8 production in human polymorphonuclear leukocytes (PMNs) and mononuclear cells (MNCs) [60]. The cytokine IL-8 release is affected by *Bifidobacterium* spp. production of soluble anti-inflammatory factor IкB-ζ which inhibit NF-кB mediated IL-8 expression [59, 61]. Therefore, *B. bifidum* BGN4 [pBESIL10] and *B. bifidum* BGN4 [pBES2] CFCS significantly decreased the IL-8 release in HT-29 cells, since both are *B. bifidum* BGN4.

*B. bifidum* BGN4 [pBESIL10] is a bacterium with superior intestinal adhesion ability when compared to other recombinant bacteria [14], and it also has immunosuppression effects on macrophage cells [15]. In addition, the shuttle vector pBES2, which can be transformed into *E. coli* and *Bifidobacterium* [41], and the *gap* promoter [46, 62] with a signal peptide (*bbif_1761)* [62, 63] which had high expression and excellent secretion ability from *B. bifidum* were used. Therefore, IL-10 may be more effective in the large intestine when delivered by this engineered bacterium versus those employed in previous studies. Also, in terms of detecting the amount of secreted human IL-10 with ELISA, we confirm that the *B. bifidum* BGN4 [pBESIL10] is able to produce and secrete higher hIL-10 levels than those previously reported for recombinant *Bifidobacteria* and *L. lactis*. In our study, we observed a consistent production of hIL-10 comparable or even greater than those described with other plasmids with transformed probiotics, with a yield of 1473 ± 300 ng/mL of hIL-10. While conducting animal cell experiments, we were able to confirm the benefits of hIL-10 and the efficacies of *B. bifidum* BGN4 supernatant. Human IL-10 secreted by *B. bifidum* BGN4 [pBESIL10] into the supernatant significantly inhibited IL-6 production in RAW 264.7 cells, whereas the B. bifidum BGN4 supernatant did not affect IL-6 production. IL-10 does not stimulate or affect IL-8 production by LPS or TNFα induced by human colon adenocarcinoma HT-29 cells, however *Bifidobacterium* sp. inhibits IL-8 release in LPS-treated HT-29 cells.

Although the efficacies of recombinant bacteria through animal cells have been confirmed in this study, further studies are needed to determine whether the effect on the human body is significant using animal models and clinical experiments in vivo. Since cytokine IL-10 and *B. bifidum* BGN4 have been applied in various fields, they have also shown potential in treating human autoimmune diseases such as inflammatory bowel disease (IBD) [32, 33], cancer, rheumatoid arthritis [28, 33], and in the prevention of obesity and allergies [12]. Therefore, recombinant *B. bifidum* BGN4 [pBESIL10] can potentially be widely used. In addition, considering that the ecological activity area of *L. lactis* is the small intestine, while *Bifidobacterium* is largely resident in the large intestine, it is expected that synergic functional effects will occur when *Lactococcus* and *Bifidobacterium* are applied together as hosts [48]. Furthermore, the IL-27 with Blimp-1 could induce IL-10 in CD4 T cells downstream of STAT1/STAT3 signaling, resulting in a broader immunosuppressive response [39, 64].

## Conclusion

The objective of this study was the production and evaluation of a recombinant *B. bifidum* BGN4 which could deliver human IL-10 to the host intestines. As a plasmid construction process, a *gap* promoter and signal peptide from *Bifidobacterium* spp. and the human IL-10 gene optimized to *Bifidobacterium* were cloned into the *E. coli-Bifidobacterium* shuttle vector pBES2. More than 1473 ± 300 ng/mL of human IL-10 was detected in the cell free culture supernatant of *B. bifidum* BGN4 [pBESIL10]. *B. bifidum* BGN4 [pBESIL10] efficiently produces and secretes significant amounts of biologically active human IL-10, and its production level is the highest of all human IL-10 production reported to the academic community to date. Further in vivo and clinical research should be pursued to evaluate therapeutic properties of *B. bifidum* BGN4 [pBESIL10] synthesizing IL-10 as a treatment for various inflammation-related diseases, including inflammatory bowel disease, rheumatoid arthritis, allergic asthma, and cancer immunotherapy.

## Data Availability

The datasets used and/or analysed during the current study are available from the corresponding author on reasonable request.
